# Supplementary material for: Left ventricular long-axis function in hypertrophic cardiomyopathy - Relationships between e`, early diastolic excursion and duration, and systolic excursion
Source: PLoS One. 2020 Oct 7;15(10):e0240296. doi: 10.1371/journal.pone.0240296 (PMC7540873; doi:10.1371/journal.pone.0240296)
Supplement: Attachment — Submitted filename: Response to reviewers August 2020.docx [file pone.0240296.s002.docx]

**RESPONSE TO REVIEWERS**

We thank the reviewers for their constructive suggestions and have addressed the issues they have raised raised below.

Reviewer #1: The manuscript by Peverill et al represents a case-control study with the purpose to highlight the differences of a wide range of LV TDI indices in HCM conpared to healthy volunteers. Overall, it's a well written manuscript which analytically sheds some light to the interrelations of the TDI parameters that reflect the LV long axis function in this common cardiomyopathy. In my opinion, however, there are some concerns.

1) Although the statistics do not seem compromised, the study sample is too small even for a rare cardiomyopathy. Moreover, all HCM patients had asymmetrical septal hypertrophy, which is the most common type of HCM, but it does not represent the broad spectrum of phenotypic diversity that characterizes the cardiomyopathy. To lend more support only 2 patients (9%) had non-obstructive phenotype and this is not the case in HCM's general population. Therefore, the results of the study cannot be extrapolated to the general HCM population. This limitation should be clearly stated.

*# Although the present study can be considered small, it is important that it was already well known that there are substantial differences in LV long-axis TDI findings, and in particular e`, between HCM and age and sex matched control subjects. Large numbers were therefore not going to be required to compare long-axis LV function in HCM subjects with a well-matched control group, and indeed the size of the two groups in the present study proved sufficient to test the study hypotheses. There are therefore no concerns about type 2 error with respect to the main aims of the study. We agree that our findings can only be said to apply to the most common HCM phenotype of asymmetrical septal hypertrophy and there are now additional statements in the limitations paragraph in the discussion with respect to this. Included now are statements about the large variability in the HCM phenotype, the specific phenotypic features of our subject group, and that our results cannot be assumed to reflect long-axis function in subjects with other HCM variants.*

2) The discussion section is too long to maintain the reader's interest. I understand that the results are too many, however, the authors should highlight the most important which may have clinical implications.

*# A number of less important sections in the Discussion have been removed.*

3) Throughout the manuscript there are long sentences which lose their meaning. The authors should revise these throughout the manuscript.

*# A number of the longer sentences have been changed.*

4) The most important concern, however, is that the authors state that the need for informed consent was waived as all analyses were performed on anonymized data. However, in the data supporting document at the second sheet and second column there are surnames. Could you explain this discrepancy? If these are the surnames of patients the lack of consents raises serious ethical concerns. In any case these surnames should be removed.

*# We agree that this was a most concerning oversight. The spreadsheet has been edited to remove the worksheet with identifying data.*

Reviewer #2: This is interesting data reframing old and offering new insights into hypertrophic cardiomyopathy.

The Background in the Abstract talks about e' being a measure of LV dysfunction - but shouldn't that be "diastolic" dysfunction?

*# We agree that e` has been commonly thought of as a measure of diastolic function. However, given increasing evidence that e` is determined in part by the previous contraction (see supporting references in manuscript), and that one of the main aims of the present study was to investigate the determinants of e` and EDExc in HCM, the more general term of dysfunction seemed appropriate.*

It is known that LVET is prolonged with high pressure gradients in HOCM and I am surprised they did not find a relationship between SDur and the gradient. Or did they? Was there any difference between those with significant gradients vs those without?

*# HCM was associated with prolongation of lateral SDur, but not septal SDur, in the present study. There were no differences in either septal or lateral SDur based on comparison of those HCM subjects with a resting versus those without a resting LV outflow tract gradient >30 mmHg. However, this analysis has not been included in the Results as it was not the focus of the current study, and we also had concern about the possibility of a type 2 error. A previous report of a correlation of LVET (corrected using the square root of the heart rate) with LV-aortic pressure gradient was based on simultaneous invasive measurements of gradient and LVET (Wigle et al Circulation 1967). It is therefore possible that simultaneous gradient and TDI acquisition may be necessary to accurately detect a relationship of the gradient with SDur.*

How did the extent of hypertrophy within the HCM patients affect the data? Did basal hypertrophy have less abnormalities than those with marked hypertrophy?

*# This interesting question has been considered in several ways, but because of concern about the possibility of a false negative finding we have not included all of the analyses in the Results, and have not mentioned this issue in the Discussion. The percentage differences in the average e`, EDExc and EDDur between the HCM and control subjects for both septal and lateral walls are now included in the Results. Although the percentage differences in e` and EDExc between the groups were not identical for the two walls (40% v 55% lower for septal and lateral e`, and 25% v 33% lower for septal and lateral EDExc, respectively), neither were they substantially different. The following regression analyses were also performed but have not been included in the manuscript. In HCM subjects there were correlations between septal and lateral e` (r=0.81, p<0.001), septal and lateral EDExc (r=0.66, p=0.001), and septal and lateral EDDur (r=0.64, p=0.001). There were no correlations of septal wall thickness with septal e`, EDExc or EDDur, with or without adjustment for the relevant lateral wall value of these variables (p>0.1 for all). Together the above findings are consistent with an effect of HCM on the long-axis function of both LV walls which is not dependent on variability in wall thickness, and they do not support an additional effect on long-axis function from the degree of septal wall thickening. However, neither do we feel justified in saying we can exclude such an effect.*

*Our findings with regard to septal and lateral e` can be compared to those of the cited article by Nagueh et al (Circulation, 2001). Their HCM group had similar septal and posterior wall thickness to our HCM group. Their results showed similar percentage differences between HCM and control subjects for the average e` of the septal and lateral walls (63% lower septal e` and 65% lower lateral e` in HCM subjects), and thus also did not support a major effect of variability in asymmetric septal wall thickness on LV long-axis function.*

In that vein, the first sentence mentions that HCM is a "hereditary heart disease of dominant inheritance". Actually only about a third of patients have a dominant hereditary background. That should be corrected.

*#The statement regarding dominant inheritance has been removed.*

It would have been nice to look at E/e' and other parameters as this is used clinically.

*# E/septal e`, E/lateral e` and E/average e` have now been included in Table 2. None of these variables were normally distributed and they have been presented as median [range]and analysis was performed with a Mann-Whitney test. A sentence about the increased magnitude of these variables in HCM subjects is now included in the text of the Results. In the Methods, a reference is cited for why E/e` cannot be used to accurately estimate mean left atrial pressure in HCM.*

IVRT shortens with increased left atrial pressure and that should have been mentioned.

*# A sentence has been added to what is now the 5th paragraph in the discussion regarding the possibility of the effects of left atrial pressure on IVRT with a relevant citation.*

I am not surprised that e' and EDExc are related because they are different ways of measuring the same "wave".

*# As the reviewer suggests, a correlation between e` and EDExc is not surprising, and it was also expected based on previous findings in healthy adult subjects. On the other hand, to our knowledge EDExc had not been specifically investigated in HCM, the relationship of e` with EDExc in HCM has not been investigated, and one of the main aims of this study was to determine if e` in HCM might also be influenced by prolongation of EDDur. A new finding of this study was that prolongation of EDDur did occur in HCM and was an important determinant of the lower e` in HCM in conjunction with the lower EDExc in HCM.*

It would have been useful, again for clinical purposes, to look at pulmonary vein pulsed Doppler profile and see how that correlates.

*# Pulmonary vein flow assessment was not routinely performed in the cohort.*

On a personal note, I think the discussion is way too long and should be condensed and eliminate most of the speculation.

*# The discussion has been reduced in length.*
