# Supplementary material for: Left ventricular long-axis function in hypertrophic cardiomyopathy - Relationships between e`, early diastolic excursion and duration, and systolic excursion
Source: PLoS One. 2020 Oct 7;15(10):e0240296. doi: 10.1371/journal.pone.0240296 (PMC7540873; doi:10.1371/journal.pone.0240296)
Supplement: Attachment — Submitted filename: Response to reviewers 2 September 2020.docx [file pone.0240296.s003.docx]

RESPONSE TO REVIEWERS

We thank the reviewers for their further comments and are very pleased to see hear that Reviewer 1 believes it is satisfactory in its current form. The issues raised by Reviewer 2 are addressed below.

Reviewer #1: The manuscript was improved substantially. All my comments were addressed sufficiently and the paper is now publishable.

Reviewer #2: I like the article. It makes one think about the relationship between systolic and diastolic excursion. But I still have the following minor concerns about the revised version.

*# 1. Abstract - "(e') is recognized to be a measure of left ventricular (LV) dysfunction.." Actually it is recognized to be a measure of diastolic dysfunction by most people. While this paper attempts to challenge this notion, for most people this is not considered accurate. I am not content this was not changed or acknowledged.*

The authors are concerned with the use of "recognized" together with e` and diastolic dysfunction in that sentence as the word "recognized" implies acknowledgment of the validity of something which is being challenged in the present study. In an attempt to address the reviewer's issue but also avoid the use of "recognized" the first sentence of the abstract has been changed to "The peak velocity of early diastolic mitral annular motion (e`) is believed to provide sensitive detection of left ventricular (LV) diastolic dysfunction in hypertrophic cardiomyopathy..."

*# 2. Introduction - first sentence - says "..is a hereditary heart disease.." - this is only true about 35% of the time. I am not content this was not changed to reflect this fact.*

The authors agree that "hereditary heart disease" can be considered inappropriate as it implies that it has been inherited in all cases. "Hereditary" has been changed to the more accurate term of "heritable", which is the term that was used in the cited reference from Ommen et al.

*# 3. Table 4 makes no sense. Whereas Table 5 lists the dependent and independent variables correctly, this is not stated for Table 4. HCM per se is not an independent variable.*

The authors do not accept that HCM cannot be described as an independent variable in the manner it has been used in the analysis shown in Table 4. The methods used in the analysis are described in the Statistics session in the following sentences. "Multivariate linear regression analysis in the combined HCM and control group was performed to assess the relationships between long-axis systolic and diastolic variables. HCM versus control was included as a dummy variable (1 v 0) as a last step in multivariate models to see if it accounted for further variance in the dependent variable after inclusion of the other independent variables." Testing of the contribution of a categorical variable by including it as a dummy variable in multivariate linear regression analysis is a recognized technique (see section on Categorical Predictor Variables in review by Slinker et al. Multiple linear regression: Accounting for multiple simultaneous determinants of a continuous dependent variable. Circulation 2008; 117: 1732-1737). If there is ongoing concern about this issue then the authors would be pleased for the editor to obtain independent statistical advice.
